# Cardiovascular Aging and Risk Assessment: How Multimodality Imaging Can Help

**DOI:** 10.3390/diagnostics14171947

**Published:** 2024-09-03

**Authors:** Maja Hrabak Paar, Miroslav Muršić, Jens Bremerich, Tobias Heye

**Affiliations:** 1 Department of Diagnostic and Interventional Radiology, University Hospital Center Zagreb, Kispaticeva 12, HR-10000 Zagreb, Croatia; 2 Clinic of Radiology and Nuclear Medicine, University of Basel Hospital, Petersgraben 4, CH-4031 Basel, Switzerland

**Keywords:** cardiovascular risk, aging, ultrasonography, computed tomography, magnetic resonance imaging

## Abstract

Aging affects the cardiovascular system, and this process may be accelerated in individuals with cardiovascular risk factors. The main vascular changes include arterial wall thickening, calcification, and stiffening, together with aortic dilatation and elongation. With aging, we can observe left ventricular hypertrophy with myocardial fibrosis and left atrial dilatation. These changes may lead to heart failure and atrial fibrillation. Using multimodality imaging, including ultrasound, computed tomography (CT), and magnetic resonance imaging, it is possible to detect these changes. Additionally, multimodality imaging, mainly via CT measurements of coronary artery calcium or ultrasound carotid intima-media thickness, enables advanced cardiovascular risk stratification and helps in decision-making about preventive strategies. The focus of this manuscript is to briefly review cardiovascular changes that occur with aging, as well as to describe how multimodality imaging may be used for the assessment of these changes and risk stratification of asymptomatic individuals.

## 1. Introduction

Cardiovascular diseases (CVDs) are a major cause of disability, premature death, and escalating healthcare costs throughout the world. Advancing age is a major and non-changeable risk factor for cardiovascular diseases, partly because advanced age increases the chances of developing cardiovascular risk factors. Cardiovascular aging is a result of genetics, with an interplay between modifiable epigenetic, lifestyle, and environmental factors [1]. Therefore, many people achieve an old age without evidence of these diseases, whereas a significant number of younger people suffer from them. Since cardiovascular diseases are frequently silent until major fatal or non-fatal cardiovascular events occur, substantial investments have been made in the development of tools to detect individuals with “old” arteries who are at a high risk for developing coronary artery disease and atherosclerotic disease at other vascular sites. Cardiovascular age is the predicted age of a person’s vascular system based on the person’s CVD risk factor profile. It is calculated by identifying the chronological age of a person with the same predicted CVD risk but normal levels of CVD risk factors [2]. In individuals at a high risk, effective management of modifiable risk factors through lifestyle changes and pharmacotherapy can significantly reduce cardiovascular morbidity and mortality [3].

The concept of cardiovascular aging is also important because of the changing demographics in Western societies, with a constantly increasing aging population and a high prevalence of risk factors, causing premature cardiovascular aging in people as young as 30, especially males [4]. Between 2015 and 2050, the proportion of the world’s population over 60 years will nearly double, from 12% to 22% [5]. The most important molecular hallmarks of cardiovascular aging include disabled macroautophagy, loss of proteostasis, genomic instability, epigenetic alterations, mitochondrial dysfunction, cell senescence, dysregulated neurohormonal signaling, and inflammation [6]. These processes lead to morphological and functional changes in the aging cardiovascular system, characterized mainly by wall thickening, stiffening, and dilatation of large arteries, left ventricular (LV) hypertrophy, myocardial fibrosis, diastolic dysfunction, valvular degeneration, and atherosclerosis.

Multimodality imaging has an increasing role in tracking cardiovascular aging [7]. Echocardiography and magnetic resonance imaging (MRI) can both be used to assess cardiac morphology and function, as well as aortic stiffness. The significant advantage of cardiac MRI is its tissue characterization capability, allowing for the detection and quantification of myocardial fibrosis. Ultrasonography is the first-line modality for the assessment of carotid artery wall changes. Coronary artery calcium (CAC) scoring using computed tomography (CT) may be used for advanced cardiovascular risk stratification, mainly in individuals close to the treatment decision thresholds. Coronary CT angiography has become the first-line imaging modality for patients with suspected chronic coronary syndrome [8,9].

This manuscript aims to review basic concepts of cardiovascular aging and age-related cardiovascular diseases, with a special emphasis on the multimodality imaging evaluation of cardiac and vascular aging, as well as CVD risk assessment.

## 2. Vascular Aging

**Figure 1:**
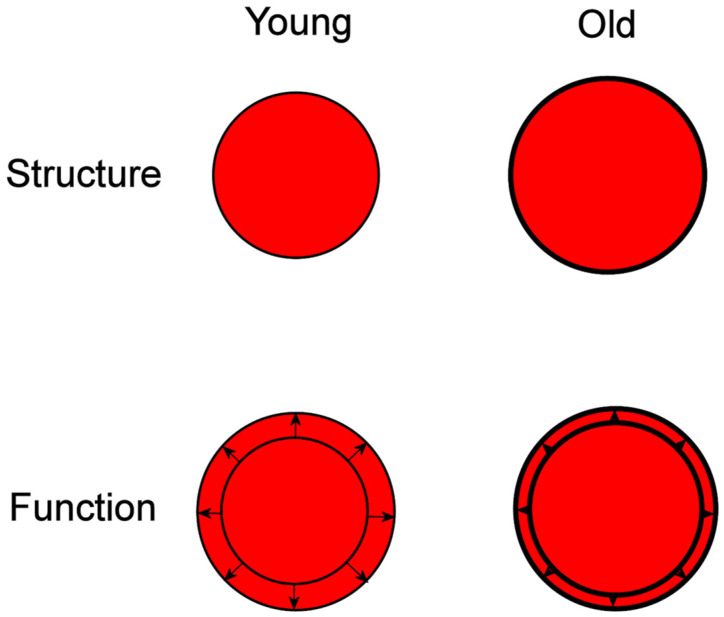
Structural and functional differences between young and the old aortas. Structural changes occurring with aging include aortic lumen dilatation and wall thickening. Functional changes are characterized by aortic wall stiffening, leading to reduced wall compliance (arrows and arrowheads) and decreased difference between end-diastolic (inner circle) and end-systolic (outer circle) cross-sectional aortic areas in older individuals.

Vascular changes are inevitable and ubiquitous during the aging process. Vascular aging can occur to varying extents depending on intrinsic arterial properties, as well as on the presence or absence of risk factors. It is mainly characterized by structural remodeling of large elastic arteries, with wall thickening and dilatation being the most prominent among them, as well as by functional impairment due to arterial stiffening (Figure 1). Arterial wall thickening and aortic stiffening are independent predictors for the new onset of arterial hypertension, suggesting that subclinical vascular abnormalities are present even before the onset of hypertension and might have an important role in hypertension development [10].

A healthy arterial system has a large buffering capacity that converts pulsatile flow ejected from the left ventricle into a more continuous flow that enables the maintenance of organ blood supply during diastole. Elastic arteries distend during systole, acting as an elastic blood reservoir, and recoil again during diastole. This process is known as the Windkessel effect, analogously to the role of an air chamber that maintains a steady flow through the spout in historical fire engines. Measurement of the arterial elasticity at the level of the aorta is reasonable, since the thoracic and abdominal aorta provide the largest contributions to the arterial buffering function [11].

“Old” arteries are not a synonym for subclinical atherosclerosis, but they are a “fertile ground” for the development of cardiovascular diseases, and they lower the threshold for clinically significant signs and symptoms of these diseases [12]. Unsuccessful vascular aging precedes clinically apparent disease and is related to a higher risk for the development of atherosclerosis, hypertension, and stroke [13]. The positive impact of aerobic exercise on arterial stiffness and vascular age has been demonstrated in numerous studies [14,15,16]. 

Wall thickening is mainly the result of an age-associated increase in vascular smooth muscle cell (VSMC) media-to-intima migration with consequent progressive intimal infiltration, as well as medial hypertrophy/hyperplasia. Arterial wall thickening is clinically mostly assessed via ultrasounds of intima-media thickness (IMT) of the common carotid artery.

Dilatation and elongation of the aorta are a consequence of fatigue and fracture of elastin fibers, which, together with repetitive pulsatile strain, lead to arterial remodeling. The increased content of elastin fibers and greater extent of stretch could be an explanation for why dilatation and elongation are most pronounced in the ascending aorta, with relative sparing of peripheral muscular arteries [17,18]. According to Laplace’s law, the dilatation of large arteries increases vascular wall tension and further promotes oxidative stress and atherogenesis. Two-fold elongation of the ascending aorta between 20 and 80 years of age without accompanying changes in the length of the descending aorta, carotid, iliac, and femoral arteries has been described in the literature [19]. Gradual luminal dilatation and reduction of vessel compliance are a part of the normal aortic aging process [20]. 

Arterial stiffening decreases the flexibility of the aorta and large arteries. This occurs due to structural changes in the vascular media, such as an increase in collagen, a reduction and fractures in elastin, and calcification, along with dysregulation of VSMC tone. As the aortic wall becomes stiffer, central systolic arterial pressure rises, diastolic arterial pressure drops, and pulse pressure increases for a given LV stroke volume. This type of arterial hypertension is known as isolated systolic hypertension and is common in older individuals. Aortic stiffening has a negative impact on the cardiovascular system, because systolic hypertension leads to LV hypertrophy, increased myocardial oxygen demand, diastolic dysfunction, and accelerated atherosclerosis, whereas decreased diastolic pressure results in decreased organ perfusion. Extension of pulsations into small arterial vessels causes small vessel degeneration in the brain and kidneys, resulting in intellectual deterioration and renal failure [21]. Arterial wall mechanics can further be adversely affected by Mönckeberg medial sclerosis, characterized by medial calcification and mostly seen in patients with type II diabetes or chronic kidney disease [22]. Also, the progression of aortic stiffness is associated with the progression of coronary atherosclerosis [23].

Arterial stiffening can be measured at the systemic, regional, and local levels [24]. The regional aortic stiffness can be estimated based on the evaluation of the pulse wave velocity (PWV) spreading through the aorta. The local stiffness is based on the measurement of the aortic cross-sectional area in diastole and systole, with calculations of aortic strain and distensibility. The systemic arterial stiffness can only be estimated based on models of the body’s circulation. Arterial stiffening leads to a reduction in arterial strain, compliance, and distensibility, as well as increases PWV. A pulse wave is a pressure wave that occurs in the arterial wall and travels along it, with its velocity determined based on the biomechanical properties of the aorta—meaning that a stiffer aorta results in a higher velocity. Carotid–femoral PWV is regarded as the “gold standard” for measuring overall aortic stiffness. An increased PWV is a well-established indicator of arterial stiffening and is considered one of the most reliable biomarkers for assessing an individual’s prospective cardiovascular and mortality risk [25]. PWV can be measured directly by determining the transit time of the pressure waveform between probes or cuffs placed on the carotid and femoral arteries, or it can be estimated from flow waveforms recorded via Doppler echocardiography or magnetic resonance imaging [26]. PWV is calculated by dividing an estimate of the distance between the common carotid artery and the femoral artery (Δx) by the time delay between the waveforms at these two locations (Δt). A carotid–femoral PWV greater than 10 m/s indicates hypertension-mediated organ damage and is used for risk assessment in hypertensive patients [27]. 

In large epidemiological studies, PWV is mostly measured using commercially available non-invasive measurement devices, whereas changes in the aortic cross-sectional area are usually assessed via echocardiography. However, none of these techniques have been recommended for cardiovascular risk assessment in asymptomatic adults outside of research settings due to measurement difficulties and substantial publication bias [3,28]. The normal PWV values from different investigations are listed in Table 1.

## 3. Cardiac Aging

Continuous structural and functional alterations of the heart occur with aging, which can finally result in a diseased heart. Due to necrosis and apoptosis, myocyte number decreases with age. In spite of this, age-related progressive LV wall thickening is present in both genders because of the enlargement of persistent myocytes [33]. Myocyte hypertrophy requires an increase in surrounding architecture composed of connective (fibrous) tissue with capillary and nerve networks that lead to further myocardial age-associated thickening.

**Figure 2:**
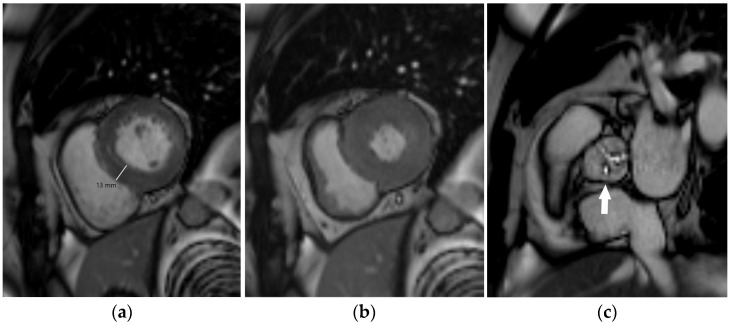
Cardiac MRI showing concentric left ventricular hypertrophy in an 80-year-old male patient with severe aortic stenosis. End-diastolic (**a**) and end-systolic (**b**) short-axis steady-state free precession (SSFP) images representing increased myocardial thickness (13 mm in end-diastole). SSFP image parallel to the aortic valve plane showing its impaired systolic opening (arrow, (**c**)).

Left ventricular hypertrophy is an adaptive remodeling process aiming to reduce LV wall stress as a response to pressure overload caused by increased arterial stiffness, systemic hypertension, or aortic stenosis. LV hypertrophy is asymptomatic for many years, but over time, the adaptive process becomes maladaptive, with an increasing risk of developing cardiovascular diseases including coronary artery disease, sudden death, and heart failure (HF). LV hypertrophy can be detected electrocardiographically, but echocardiography and MRI are more sensitive methods that also enable quantification of the LV mass (Figure 2). LV hypertrophy is considered to be target-organ damage in a hypertensive population [27], and regression of LV hypertrophy is advised as a surrogate endpoint in the treatment of hypertensive heart disease [34].

Myocardial fibrosis may serve as a cause of arrhythmia and induce ventricular wall stiffening with a progressive decrease in LV diastolic function, starting from the age of 20 years [35,36]. LV diastolic dysfunction is further fostered by age-associated changes in intracellular calcium transit; i.e., by slower calcium re-uptake from the cytosol into the sarcoplasmic reticulum after systole. Increased diastolic filling pressure leads to left atrial (LA) dilation that predisposes the heart to atrial fibrillation (AF). Progressive diastolic dysfunction is not accompanied by the change in the LV end-diastolic volume (EDV) or ejection fraction (EF) during rest. However, older people have greater EDV and achieve lower maximum EF during exhaustive exercise due to a diminished ability to reduce end-systolic volume (ESV) compared to their younger counterparts. Altogether, cardiac age-related adaptation to exercise includes greater EDV reserve, reduced ESV reserve, and preserved stroke volume over a wide range of exercise levels because of the increased use of the Frank–Starling mechanism [35]. Senescent people also achieve a lower maximum heart rate during exercise due to deficient sympathetic modulation; therefore, maximum cardiac output reserve decreases by about 30% between the ages of 20 and 85 years [35]. 

Heart conditions that emerge with advancing age include heart failure and atrial fibrillation [35]. Calcification of coronary artery plaques is also prevalent with aging. As age increases, there is a tendency for cardiac valves to thicken, stiffen, degenerate, calcify, and experience diminished repair mechanisms. The most common valve-related heart conditions associated with aging include aortic stenosis, mitral regurgitation, and aortic regurgitation [7]. In the aging heart, both supraventricular and ventricular arrhythmias are more prevalent and more complex [35].

Heart failure is the inability of the heart to keep up with the demands of the body’s tissues and to pump blood with normal efficiency. A decrease in cardiac functional reserve is a part of aging, and it is influenced by diastolic dysfunction and a limited ability to increase the heart rate during exercise. HF can exist with preserved systolic but impaired diastolic function, or also with a decreased ejection fraction. Cardiac amyloidosis is an underdiagnosed reason for diastolic dysfunction in older adults, partially due to a lack of disease awareness and also a lower sensitivity of echocardiography for diagnosing early-stage cardiac amyloidosis [37]. Table 2 summarizes the main morphological characteristics of cardiovascular aging and their imaging features.

Atrial fibrillation is the most common type of arrhythmia, characterized by electrical signals beginning in the pulmonary veins or a part of the atria outside of the sinoatrial node. It can be paroxysmal, with episodes that come and go intermittently; persistent, where it does not self-terminate within seven days; or permanent, where the patient and clinician jointly decide to stop pursuing rhythm control. Individuals older than 40 years have a 25% lifetime risk of developing AF [38]. AF eliminates atrial systolic contribution to LV filling, increasing the risk of HF in patients with compromised LV diastolic function. AF is a leading risk factor for stroke due to possible clot formation and migration from the appendage of an inefficiently contracting left atrium. At higher risk for AF are individuals with LA enlargement. In patients with AF, cardiac imaging in terms of transesophageal echocardiography, multidetector row CT, and MRI are used for the characterization of LA morphology, as well as for identification of the underlying structural heart disease that predisposes LA to adverse remodeling. The image quality of CT and MRI examinations in patients with AF can be impaired because of arrhythmia. Patients with AF frequently have dilatation of the LA vestibule or the pulmonary veins.

## 4. Standard Cardiovascular Risk Assessment

A significant proportion of the adult population without any clinical signs or symptoms of cardiovascular disease has pre-existing atherosclerotic plaques. Therefore, it is important to identify individuals in a healthy population who could have subclinical atherosclerosis and who would benefit the most from primary prevention. 

Many tools have been developed by major medical organizations for risk assessment in asymptomatic adults. Standard tools for assessing cardiovascular risk rely on data that primary care providers can easily gather during routine clinical practice, primarily including age, gender, total cholesterol, high-density and low-density lipoprotein cholesterol levels, systolic blood pressure, diabetes mellitus status, and current smoking status. Using these data, individual risk scores can be estimated using various readily accessible charts, paper scoring sheets, websites, and downloadable applications [3]. All assessment tools agree that age and gender are the most powerful risk factors. The combination of intrinsic, stochastic, and environmental factors affecting myocardial and vascular aging leads to varying development and progression of cardiovascular disease [39]. Since individual risk factors have additive or even synergistic cumulative effects, the calculation of absolute cardiovascular risk based on the combined effects of multiple risk factors is preferred over the risk estimated by the use of individual risk factors. Some examples of the most widely used tools for multi-factorial risk estimation are the Framingham risk system, the updated Systematic Coronary Risk Evaluation (SCORE2) charts, and the World Health Organization (WHO)/International Society of Hypertension (ISH) risk charts [3,40].

Atherosclerosis develops over decades, so a life-course perspective on risk assessment and prevention must be taken. In adults older than 40 years of age, an absolute 10-year risk estimation should be estimated [3,28]. Although CVD risk predictions are likely to be less accurate for individuals under 40 years old, it is still advisable to evaluate traditional CVD risk factors at least every 4 to 6 years for adults aged 20 to 39 [3,28]. Based on the risk assessment results, recommendations for clinical management of different risk factors are made. Awareness among clinicians of the current guidelines, as well as their adherence to them, is essential for a substantial reduction in mortality and morbidity due to cardiovascular diseases.

Using risk assessment tools, it is possible to estimate the 10-year risk for developing a first atherosclerotic cardiovascular disease event (nonfatal myocardial infarction, death from coronary artery disease, fatal or nonfatal stroke events) and to promote lifestyle changes with or without pharmacological preventive interventions (lipid-lowering therapy, antihypertensive therapy, nicotine replacement and/or low-dose aspirin) among patients assessed to be at higher risk. Based on the 2021 ESC Guidelines for cardiovascular disease prevention in clinical practice, individuals can be divided into three CVD risk categories [3]. For individuals with a risk close to a decision threshold, advanced risk assessment strategies including imaging techniques may be used for risk reclassification in order to identify individuals at higher risk who would benefit from primary prevention. In addition, there are female-specific cardiovascular risk factors such as the age of menarche and menopause, polycystic ovary syndrome, and infertility, which may help to identify women at higher cardiovascular risk who could benefit from more intensive preventive measures [41]. In addition to tobacco smoking, it has been shown that cannabis use is also associated with adverse cardiovascular outcomes, with higher odds of adverse outcomes if cannabis is more frequently consumed [42]. Other factors that may change the individual’s cardiovascular risk include nutrition, physical activity, mental well-being, and environmental influences like air pollution [1].

## 5. Multimodality Imaging of Cardiovascular Aging with Advanced CVD Risk Assessment

Advanced methods for CVD risk estimation based on new biomarkers are continuously being developed as potential risk modifiers, including several cardiovascular imaging methods like CAC scoring, carotid IMT, or aortic stiffness that could detect preclinical vascular changes. A recent publication showed that the addition of risk-modifying characteristics on top of existing risk models can better estimate CVD event risk [43]. These risk modifiers have the potential to exceed traditional risk factors, but at present, they cannot be accepted for routine screening risk assessment in asymptomatic adults because of high costs and lower availability. However, they should be considered for persons with uncertain treatment decisions after a traditional risk assessment and performed as a second step for deciding on preventive strategies. Compared to the CAC score, there is a tendency of vascular age overestimation by traditional risk methods in low- to intermediate-risk patients [44]. Machine learning algorithms are being tested and have the potential to further improve CVD risk prediction [45]. Table 3 summarizes the features, indications, advantages, disadvantages, and restrictions for the use of different cardiovascular imaging modalities in advanced CVD risk assessment.

### 5.1. Ultrasonography and Echocardiography

Ultrasonography can be used for the evaluation of the carotid wall (carotid IMT and plaque evaluation), assessment of cardiac morphology and function, and for measurements of aortic stiffness. 

**Figure 3:**
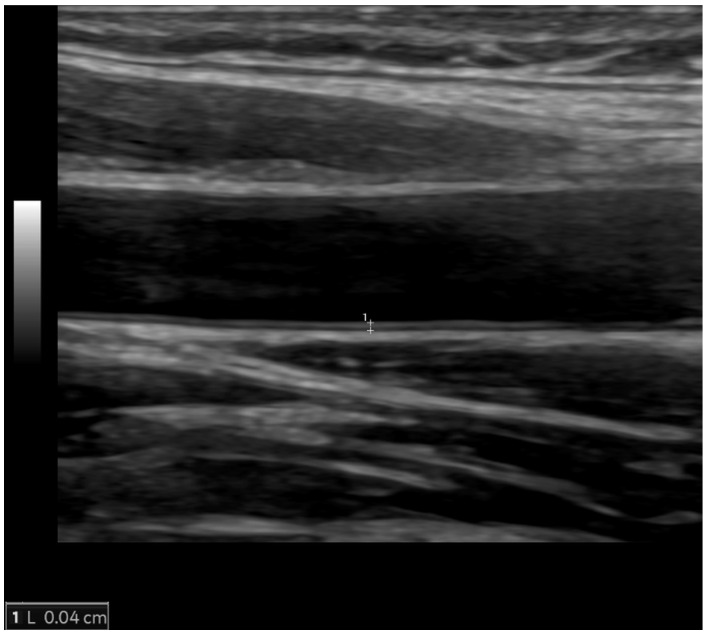
The normal value of carotid intima-media thickness of 0.4 mm (between the calipers) in a young male individual.

Carotid IMT (Figure 3) is a structural measure of vascular age that can be used for advanced cardiovascular risk assessment. It is not synonymous with atherosclerosis, but it can detect early stages of vascular aging. Carotid IMT refers to the combined thickness of the intimal and medial layers of the arterial wall, typically measured using high-resolution B-mode ultrasound on the far wall of the common carotid artery. It should be measured using a linear-array transducer that operates at a fundamental frequency of at least 7 MHz. As per the consensus from the American Society of Echocardiography, carotid IMT should be measured bilaterally at the far wall of the distal common carotid artery within a 1-cm-long straight segment just before the carotid bulb and compared with values from a normative dataset [46]. Cardiovascular risk increases progressively with rising IMT, but an IMT value of 0.9 mm is generally considered abnormal [27]. To enhance sensitivity in detecting subclinical atherosclerosis, carotid IMT measurement should be complemented by a comprehensive scan of the extracranial carotid arteries to check for the presence of carotid plaques. Atherosclerotic plaques are defined as a focal wall thickening at least 50% greater than that of the surrounding vessel wall or as a focal region with IMT greater than 1.5 mm that protrudes into the lumen and is distinct from the adjacent boundary. It is a non-invasive, relatively inexpensive test, feasible in all individuals and larger populations using portable, user-friendly ultrasound equipment after very short training of a sonographer. One of the major issues regarding carotid IMT is the standardization of measurement and the requirement for a formal educational program with hands-on training and follow-up of the examiners [46]. Compared to CAC, carotid IMT is a stronger predictor of stroke (multivariable-adjusted hazard ratio of 1.3 for IMT and 1.1 for CAC) but a weaker predictor of coronary disease (multivariable-adjusted hazard ratio of 1.2 for IMT and 2.5 for CAC) [47]. A meta-analysis of 14 population-based cohorts contributing data for 45,828 individuals showed that the addition of carotid IMT measurements to the Framingham Risk Score was associated with a small improvement in 10-year risk prediction of first-time myocardial infarction or stroke (0.8%; 95% confidence interval, 0.1–1.6%), and that this improvement is unlikely to be of clinical importance [48]. According to the current 2019 ACC/AHA Guideline on the Primary Prevention of Cardiovascular Disease and 2021 ESC Guidelines on cardiovascular disease prevention in clinical practice, it is not recommended as a risk assessment tool in clinical practice due to the lack of methodological standardization and the absence of added value of IMT in predicting future cardiovascular events [3,28]. However, carotid artery plaque assessment using ultrasonography may be considered a risk modifier when a CAC score is not feasible [3]. A carotid IMT >0.9 mm or the presence of a carotid plaque is considered to be a marker of asymptomatic target organ damage that is used for risk stratification among patients with arterial hypertension [27]. Carotid plaque and high IMT are independently associated with higher atherosclerotic cardiovascular disease risk in young adults in multivariate models [49]. Clinical tools integrating carotid IMT within global risk scoring systems are not available.

Echocardiography plays a central role in the primary assessment of heart morphology and function because of its widespread availability, reliability, and lower cost compared to MRI. Using echocardiography, it is possible to differentiate slow, normal, and accelerated heart aging patterns, which may reflect biological age and predict cardiovascular and non-cardiovascular events [50]. The limitation of echocardiography in the evaluation of age-related LV hypertrophy is that it estimates LV mass from linear measurements with the assumption that LV has the shape of a prolate spheroid. Age-related diastolic dysfunction is usually evaluated echocardiographically and is characterized by reduced early diastolic and increased late LV filling with increased contribution of atrial contraction. The function of the LA can be assessed based on an evaluation of hemodynamics at the tips of the mitral valve leaflets and the pulmonary vein ostia. In addition, echocardiography (mainly transesophageal) has the role of guiding AF therapy, including thromboembolic risk prediction, screening for thrombus formation, guiding cardioversion, and planning of LA appendage closure or radiofrequency catheter ablation [51].

Echocardiography can also be used for aortic stiffness assessment, either via PWV measurement or through the evaluation of cyclic changes in the aortic diameter and calculation of aortic diameter-derived parameters [52].

### 5.2. Computed Tomography

As a risk-assessment tool, CT is currently used mainly for CAC scoring, but it can be expected that due to the significant reduction in radiation dose required for new CT scanners, coronary CT angiography or CT aortic stiffness evaluation could also be used in the future in selected groups of asymptomatic individuals.

**Figure 4:**
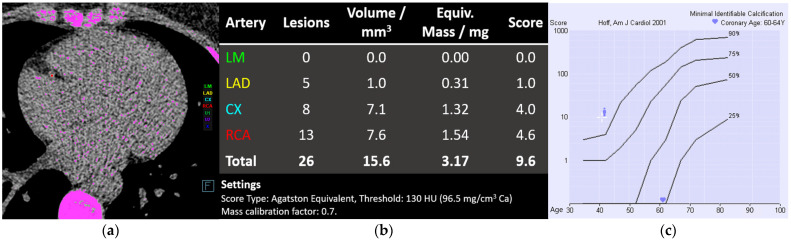
Calcium scoring in a 41-year-old female patient planned for aortic valve replacement due to bicuspid aortic valve regurgitation. All lesions with attenuation values above 130 Hounsfield units and areas ≥3 adjacent pixels (pink) in the coronary arteries are considered to be coronary calcium, the calcification of the right coronary artery (RCA) is marked in red (**a**). The results are expressed in absolute values: volume score, mass score, and Agatston calcium score (**b**). The relative risk is estimated using age- and gender-specific percentile curves showing the patient’s vascular age of 60–64 years and above the 90th percentile for her age (white cross, (**c**)).

CAC scoring was introduced in the late 1980s for electron-beam CT scanners [53], but currently, the mode for measuring coronary calcium at most centers is multidetector row CT (Figure 4). CAC scoring requires postprocessing of non-contrast-enhanced, prospectively electrocardiography (ECG)-triggered axial scans of the coronary arteries. Lesions with attenuation values above an arbitrary threshold of 130 Hounsfield units (HU) and an area ≥3 adjacent pixels (at least 1 mm^2^) are considered to be coronary calcifications [54]. The total amount of coronary calcium is calculated using dedicated, commercially available software; usually one of two widely used systems: the Agatston method or the volume method. The original Agatston score is calculated from 3 mm thick axial images as the sum of all products between the calcification area multiplied by an arbitrary density factor that depends on the X-ray attenuation coefficient (1 = 130 to 199 HU, 2 = 200 to 299 HU, 3 = 300 to 399 HU, and 4 ≥ 400 HU). Scanning with thinner slices leads to oversampling, and the partial volume effect changes the attenuation values and the density factor of a plaque, leading to different measurements. The volume score resolves the issue of slice thickness and spacing by computing a volume of voxels containing calcium, not taking into account the density of calcium. The volume values are similar to the Agatston score but have improved interscan reproducibility [55]. There is also a third “mass” method that measures the absolute value of mineral mass in milligrams and is the only quantitative measurement that directly represents the amount of coronary calcium. This method requires the use of CT calibration phantoms and is calculated as a sum of the product of the calibration factor, the volume, and the mean HU for each lesion. The mass score has improved accuracy and reproducibility but has not been widely adopted into practice or research. The original Agatston score remains the most extensively studied method in both clinical and research settings and is therefore the most widely accepted [55].

Since the CAC score increases with advancing age and is generally higher in men, the score is commonly ranked using age- and gender-stratified percentile curves of the CAC score in a reference population. The Society of Cardiovascular Computed Tomography in 2018 proposed a Coronary Artery Calcium Data and Reporting System (CAC-DRS) to create a standardized method to communicate CAC findings and to facilitate clinical decision-making, with recommendations for subsequent patient management [56].

A typical effective dose of prospectively ECG-triggered cardiac scan is low (0.9–1.1 mSv), which is lower than annual background radiation exposure [57,58]. In a phantom study, new state-of-the-art CT systems with thin-slice protocols showed increased detectability of small and low-density calcification, with a reduced radiation dose and improved intrascanner and interscanner reproducibility [59]. 

Healthy arteries do not contain any detectable calcium, so coronary calcification is highly specific for atherosclerotic plaques [60]. As calcium is a subcomponent of atheroma, the CAC score is a reasonable estimate of the total (calcified and non-calcified) coronary atherosclerotic disease burden. A negative CAC test (score = 0) makes the presence of atherosclerotic plaques and significant luminal obstructive disease highly unlikely and is consistent with a low risk (0.1% per year) of cardiovascular events. Compared to individuals with a CAC score of 0, those with any measurable calcium have a fourfold increased risk for coronary events, and higher CAC scores are associated with higher event rates [61]. The current literature supports a CAC = 0 as a strong downward CVD risk classifier in older patients, whose plaque burden predominantly involves calcified plaques (power of zero). However, a CAC = 0 does not reliably exclude obstructive coronary artery disease in patients under 40 years who might have a higher burden of non-calcified plaques [62]. CAC score typically progresses from 10–20% of the baseline value per year, and yearly, approximately 7% of persons older than 45 years who do not have coronary calcium at baseline will develop detectable coronary calcium [57]. A higher CAC score was shown to be strongly associated with long-term, all-cause mortality and a greater proportion of deaths due to cardiovascular disease and coronary heart disease [63]. Studies have shown that the total burden of atherosclerosis is a stronger CVD risk factor than a solitary focus of coronary stenosis [64].

Coronary calcification is not an indicator of stability or instability of an atherosclerotic plaque and can be found in the intima of both obstructive and nonobstructive lesions. Therefore, the presence of coronary artery calcification in asymptomatic persons does not provide a rationale for revascularization but rather for risk factor modification [54]. Several large studies showed that the CAC score is associated with the risk of future cardiovascular events, and when it is added to traditional risk factors, it results in a significant improvement in the classification of risk for the prediction of CHD events in an asymptomatic population [65,66]. CAC-derived vascular age can also improve CVD risk discrimination in the primary prevention of subjects with familial hypercholesterolemia [67]. Moreover, CAC screening enhances adherence to medical therapy and could motivate individuals to adopt beneficial behavioral or lifestyle changes [68].

CAC testing is considered to be the most useful method for advanced risk assessment among individuals who are at intermediate 10-year risk of cardiac events based on formal risk assessment, with a net reclassification improvement of 22% [69]. According to the latest European guidelines, CAC scoring may be considered to improve risk classification around treatment decision thresholds (class of recommendation IIb, level of evidence B) [3]. Similarly, the 2019 ACC/AHA Guideline on the Primary Prevention of Cardiovascular Disease states that it is reasonable to measure a CAC score to guide clinician–patient risk discussions for adults with intermediate risk (≥7.5% to <20%) or for select adults with borderline risk (5% to <7.5%) if risk-based decisions for preventive interventions remain uncertain (class of recommendation, IIa; level of evidence, B-nonrandomized) [28]. In these groups, CAC measurement can reclassify risk upward (particularly if the CAC score is ≥100 Agatston units (AU) or ≥75th age/sex/race percentile) or downward (if coronary artery calcium is zero) [28,70]. The CAC score may even refine CVD risk among lower-risk women (<7.5% 10-year risk), younger adults (<45 years of age), and older adults (≥75 years of age), but more data are needed to support its use in these subgroups [28]. An online Multi-Ethnic Study of Atherosclerosis (MESA) risk score calculator incorporating the CAC score has been created [71]. There is a growing role of routine coronary calcium assessment on all non-gated chest CTs, with or without the use of artificial intelligence and automated interpretation [64].

**Figure 5:**
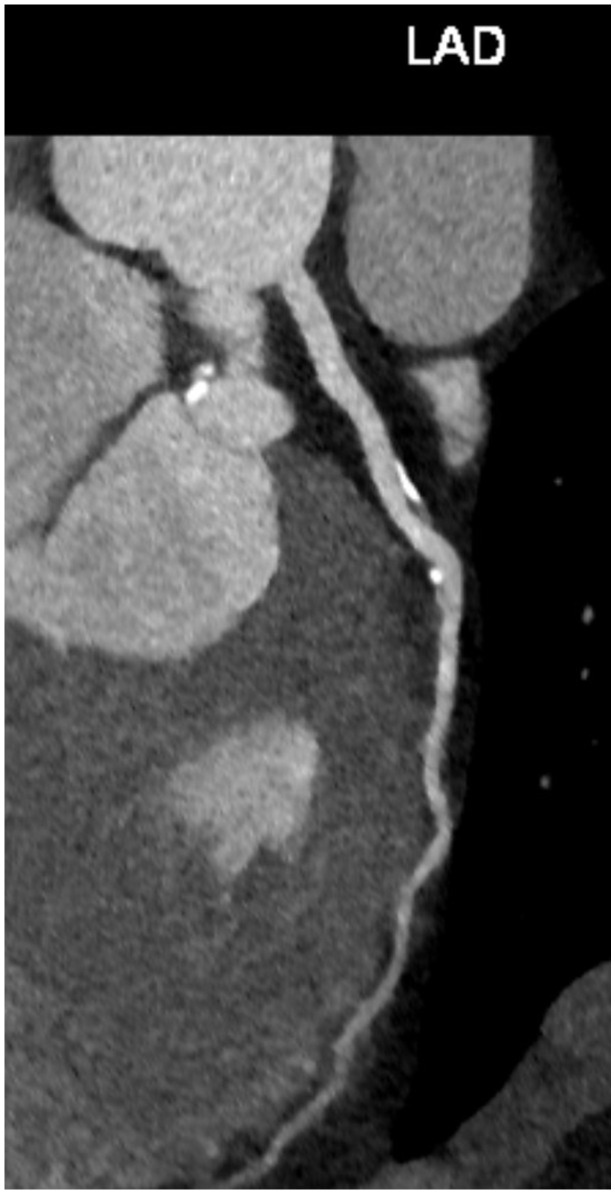
An incidental finding of left anterior descending (LAD) coronary artery plaques on a CT angiography performed in a 44-year-old male patient with a bicuspid aortic valve, dilated ascending aorta, and moderate aortic regurgitation.

Other CT manifestations of aging coronary arteries include a higher rate of intraplaque hemorrhage, rupture, and the presence of high-risk plaque features [72]. Currently, contrast-enhanced CT coronary angiography is not recommended in asymptomatic persons for the assessment of occult coronary artery disease. According to the results of the prospective multicenter international CONFIRM long-term study, there is no further incremental value offered by coronary CT angiography when its findings were added to a model incorporating CVD risk factors and CAC score [73]. However, some authors suggest that coronary CT angiography could be the best and definitive method to guide patients and physicians in the prevention of coronary artery disease because it provides additional information beyond coronary artery calcification, including the burden of all plaque types, high-risk plaque features, and in the future, the addition of machine learning and radiomic plaque characteristics (Figure 5) [74]. Additional CT applications are emerging with novel dual-energy CT (DECT) technology, including CAC scoring from virtual non-contrast imaging, better differentiation between calcified- and non-calcified plaques, more precise evaluation of high-risk plaque features, and calcium-extraction algorithms that enable more accurate stenosis quantification in the presence of calcified plaques [75]. Furthermore, using DECT, it is possible to create iodine myocardial maps that may depict myocardial perfusion defects and late-enhancing scar tissue, improving diagnostic accuracy for myocardial ischemia and infarction [75,76].

CT angiography may also be used for the assessment after surgical or interventional coronary revascularization. CT imaging of coronary stents is prone to blooming and beam-hardening artifacts obscuring the stent lumen, especially in patients with small stent diameters and thick stent struts [77]. Coronary artery bypass grafts are wider and more distant from the heart, with a low incidence of severe calcification. Therefore, they are less prone to artifacts and are ideal to be assessed via CT angiography [78]. However, native coronary arteries after revascularization are often heavily calcified, with a tendency to stenosis overestimation and challenges in visualizing distal anastomosis. These issues might also be overcome by DECT capabilities, including assessment of myocardial perfusion via iodine maps, calcium subtraction, as well as the elimination of beam-hardening artifacts produced by stents and heavy calcification using virtual monoenergetic imaging [78].

**Figure 6:**
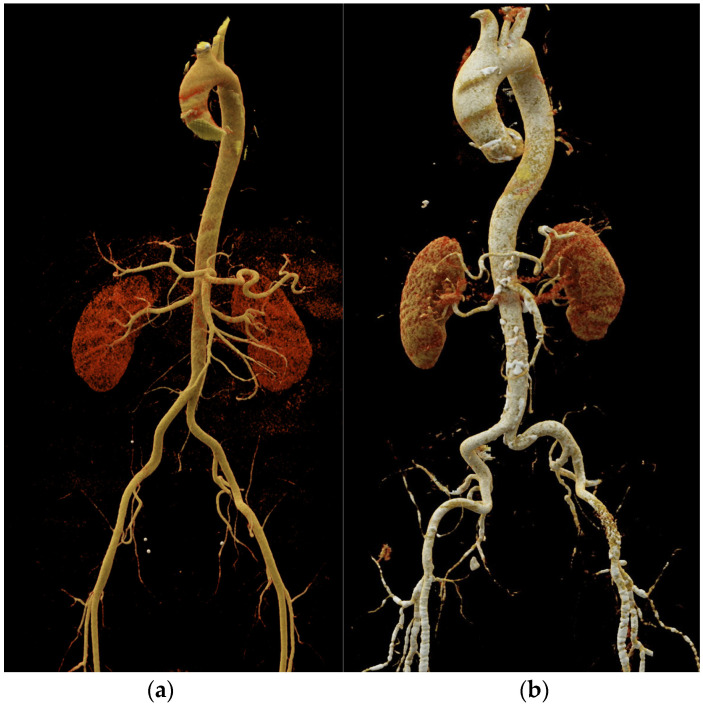
Volume rendering of a CT angiography in a 44-year-old male (**a**) and an 86-year-old male (**b**), with aging arteries become wider, tortuous, and elongated.

Additionally, aortic morphology can be detected using CT angiography, including dilatation, elongation, tortuosity, and wall thickening (Figure 6) [79]. Similarly to MRI, using CT angiography with retrospective ECG gating, it is possible to estimate local aortic stiffness via calculation of the aortic strain and distensibility [80].

### 5.3. Magnetic Resonance Imaging

MRI allows for evaluation of age-related changes in cardiac morphology and function, as well as precise measurement of regional and local aortic stiffening.

The main morphological elements of the aging heart are LV hypertrophy and myocardial fibrosis. Additionally, changes that have been detected in aging hearts using cardiovascular MRI include a decrease in ventricular volumes with normal EFs and stiffening of the myocardium and aorta [81]. MRI provides highly accurate and reproducible measures of biventricular volumes and thus is the gold standard imaging modality for assessing the ejection fraction of both ventricles in HF [82]. MRI measurement of LV mass is free of cardiac geometric assumptions and is not acoustic window-dependent, which explains its better accuracy and reproducibility compared to echocardiography [83]. Using cardiovascular MRI and radiomics features of ventricular shape and myocardial character, differences in heart aging between men and women were shown: in men, a decrease in the right ventricular volume and changes of its shape were the most important features of heart aging, whereas in women, greater sphericity of the LV and myocardium appeared more prominent [84]. A progressive decline in both vascular and myocardial tissue compliance was detected using machine learning of multiple image-derived phenotypes to predict biological age [85]. Left atrial function, assessed via cardiac MRI, might also be used as a marker of cardiovascular aging according to a community-based study of older adults without clinical CVD [86]. Based on MRI images from 18,117 healthy UK Biobank participants, an interpretable model for biological age estimation has been developed using cardiac cine MRI radiomics phenotypes, indicating that a difference between the image-based age estimates and chronological age might be an indicator of cardiovascular health and aging [87].

Myocardial fibrosis may be either generalized or localized, and a higher percentage of myocardium affected by fibrosis is correlated with a worse prognosis regarding death and cardiac events. A decline in diastolic function is a hallmark of cardiac aging, and a key driver of diastolic impairment is myocardial interstitial fibrosis and imaging biomarkers of fibrosis may predict accelerated aging [85]. Age-related diffuse accumulation of collagen within the myocardium can be difficult to identify using late gadolinium enhancement (LGE), where signal intensities must be evaluated relative to a reference region of normal myocardium. In such cases, pre- or postcontrast T1 mapping with extracellular volume (ECV) calculation is more informative than LGE. Native myocardial T1 and ECV allow for quantification of myocardial fibrosis and are increasingly used in clinical practice [88]. Using this technique, an age-related increase in the myocardial ECV was detected in both healthy human volunteers and mice [89], and age-related myocardial fibrosis was confirmed among MESA participants, with greater changes in men than in women [90]. The increase in ECV correlates with the extent of myocardial fibrosis in the histological specimens of mouse hearts [89]. Multiparametric mapping, together with LGE, is also useful for diagnosing cardiac amyloidosis, which is increasingly being recognized in older HF patients [91]. An alternative non-invasive method to confirm transthyretin cardiac amyloidosis is scintigraphy using bone-avid tracers [92].

**Figure 7:**
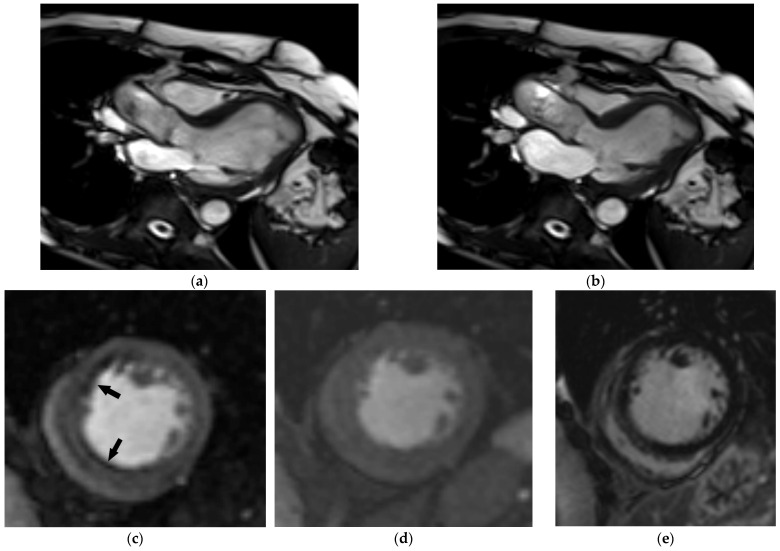
Comprehensive cardiac MRI in a 74-year-old female patient with a new onset of heart failure. End-diastolic (**a**) and end-systolic (**b**) 3-chamber view SSFP image depicting moderately impaired left ventricular systolic function (EF 31%). Midventricular short-axis adenosine-stress (**c**) and rest (**d**) perfusion image revealed stress-induced ischemia in the anterolateral and inferolateral wall (arrows) without evident late gadolinium enhancement (**e**). Coronary angiography chronic occlusion of the mid-LAD, as well as high-grade stenosis of mid-RCA and D1, were confirmed and treated.

Focal myocardial scarring can be detected using LGE, providing additional prognostic information in HF patients [93]. The distribution of LGE can provide information regarding the etiology of HF, with important therapeutic implications. This is especially the case for ischemic heart disease characterized by subendocardial LGE distribution. An additional value of MRI in ischemic heart disease is the possibility of estimating the viability of myocardium and the hemodynamic significance of coronary artery stenosis via stress imaging, which is of high importance if myocardial revascularization is planned (Figure 7). Additional caution is required during stress MRI in older people, because dipyridamole and regadenoson are more likely to cause hypotension or bradycardia when administered to older versus younger patients, and dobutamine is more likely to induce arrhythmias [37]. Other functional imaging techniques that might be used to detect myocardial ischemia are stress echocardiography, single-photon emission CT (SPECT), and positron emission tomography (PET) [8].

**Figure 8:**
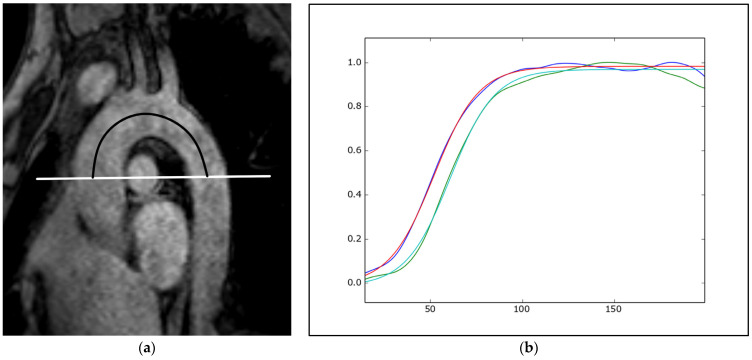
MRI assessment of pulse wave velocity (PVW). A parasagittal oblique gradient-echo image parallel to the aortic arch (**a**) is used for the distance measurement (Δx) between two sampling sites by placing a polygonal line along the centerline of the aorta (black line). The horizontal white line represents the axial plane where two-dimensional phase-contrast cine images of the ascending and descending aorta were acquired. The TT upslope method is used for transit time (Δt) measurement by minimizing the area delimited by two normalized sigmoid curves fitted to the systolic upslope of the ascending (red-blue) and descending aorta (green) velocity curves (**b**). PVW is calculated as Δx/Δt.

MRI-assessed aortic stiffness is a functional marker of vascular age and can be estimated regionally or locally. Using MRI, PWV is measured regionally, usually in the aortic arch (Figure 8). Temporal delay (Δt) can be calculated from the ascending and descending aorta flow curves acquired on two-dimensional, retrospectively gated, phase-contrast, velocity-encoded, through-plane MRI acquisitions using several methods, with the TT-upslope method showing the best performances [94]. The distance between two sampling sites (Δx) is usually measured by placing a polygonal line along the centerline of the aorta on the double-oblique sagittal prospectively gated image parallel to the aortic arch. Additionally, three-dimensional time-resolved phase-contrast (4D flow) imaging has been shown to be an accurate method for the global and segmental PWV analysis of the thoracic aorta [95]. In a longitudinal population study, a 4D flow MRI evaluation was performed at the baseline and after 6 years, showing an increase in the global PWV by 0.54 m/s (ca. 8%) compared to the mean baseline PWV of 6.6 m/s, associated with decreased helicity in the ascending aorta [96]. Proximal aortic stiffening is an early phenomenon appearing earlier than LV dysfunction and remodeling that may be detected later in life, concomitant with advanced aortic stiffness [97]. Using machine learning, it is feasible to fully automatically assess aortic rotational flow and wall shear stress from the 4D data [98].

**Figure 9:**
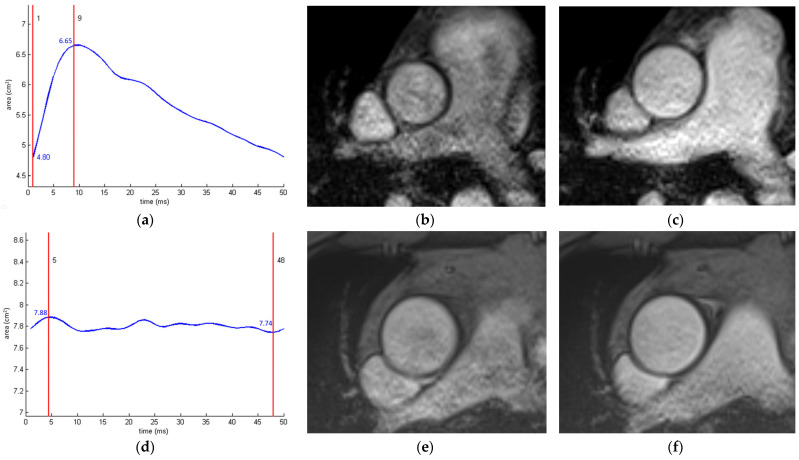
Aortic strain measurement in a young (42%, (**a**–**c**)) and an old patient (2%, (**d**–**f**)). The curves created using a validated automated software ARTerial-FUNction (INSERM UMR-S 1146) [102] represent changes in the aortic cross-sectional area throughout the cardiac cycle (**a**,**d**). End-diastolic (**b**,**e**) and end-systolic (**c**,**f**) gradient-echo cine images through the ascending aorta.

Aortic cross-sectional area-derived measures of aortic stiffness are local measures of the aortic pulsatile load (Figure 9). They are usually measured in the ascending and proximal descending aorta on retrospectively ECG-gated cine sequences. Aortic strain represents a relative cardiac cycle-dependent change in the aortic lumen area, whereas aortic distensibility exhibits relative area change per pressure step (ratio between aortic strain and pulse pressure). Pulse pressure is usually measured peripherally at the level of the brachial artery, but this measurement overestimates the pulse pressure at the site of aortic diameter measurements due to peripheral amplification of the pressure pulse as it travels from central to peripheral sites [99].

Although MRI can measure both PWV and cross-sectional area-derived aortic stiffness measures, it has been less studied than pressure- and ultrasound-based modalities in the assessment of subclinical atherosclerosis and arterial stiffness, as a more costly and less-available method. The main advantages of MRI versus other methods are the ability to assess local and regional aortic stiffness within different aortic segments and to obtain cross-sectional images in any plane. A low variability of the aortic strain and PWV measurements was shown in a multicenter trial setting using standardized MRI protocols [100]. Moreover, MRI and CT allow for exact distance measurements for PWV calculations, rather than aortic-path estimation from the body surface measurements. Therefore, MRI and CT are considered to be the gold standard for path length assessment, ideally using biplanar images for a 3D reconstruction of the centerline [101]. Despite this, these techniques are not favored as reference techniques to validate PWV measurement devices due to data quality issues and heterogeneity of acquisition protocols [101].

## 6. Conclusions

The structure and function of the cardiovascular system change with aging, and this process may be accelerated by CVD risk factors. Vascular aging is mainly characterized by the structural remodeling of large elastic arteries, with wall thickening and dilatation being the most prominent among them, as well as by functional impairment due to arterial stiffening. Heart conditions that emerge with advancing age are LV hypertrophy, myocardial fibrosis, heart failure, atrial fibrillation, and coronary artery calcification. Multimodality cardiovascular imaging allows advanced CVD risk assessment and visualization of cardiac and vascular aging changes, mainly by calculation of CAC score and evaluation of arterial wall thickness and compliance.

## 7. Future Directions

Currently, there are several approaches to CVD risk assessment recommended by the world-leading cardiovascular societies. It is expected that artificial intelligence could further improve risk assessment protocols, and that new cardiovascular imaging biomarkers with enhanced sensitivity, improved specificity, and reduced ionizing radiation will be developed for personalized CVD risk prediction and preventive strategies. Novel automatic segmentation tools allow for accurate and efficient delineation of cardiac structure, improving quantification of the cardiac structure and function [103,104,105]. Additionally, it is essential to standardize imaging biomarker acquisition and interpretation for their optimal use in research and clinical practice.

## Figures and Tables

**Table 1:** Age-specific normal pulse-wave velocity (PWV) values measured in different studies and expressed as means ± standard deviations [95% confidence interval].

Study	Parikh et al., 2016 [29] ^a^	Baier et al., 2018 [30] ^b^	Díaz et al., 2014 [31]	Parikh et al., 2016 [29] ^a^	van Hout et al., 2021 [32] ^b^
PWV technique	Vicorder device	Vicorder device	Institution-developed technique using silicon piezoresistive pressure sensors	MRI	MRI
Number of participants	80	8509	780	80	397
PWV (m/s) according to age group					
10–19 years	NA	NA	5.04 ± 0.72 [4.92–5.15]	NA	NA
20–29 years	6.7 ± 0.9	7.2 [5.0–13.1] (age range: 18–29)	5.86 ± 0.92 [5.68–6.03]	4.5 ± 1.5	NA
30–39 years	6.9 ± 1.0	7.8 [5.5–14.0]	6.32 ± 0.82 [6.16–6.47]	5.1 ± 0.8	NA
40–49 years	7.5 ± 1.2	8.9 [6.0–15.2]	6.85 ± 0.91 [6.68–7.03]	6.5 ± 1.7	5.4 [5.3–5.6] (age range: 40–49)
50–59 years	8.0 ± 1.7	9.4 [6.1–16.1]	8.15 ± 1.17 [7.97–8.33]	6.2 ± 1.6	5.8 [5.6–5.9] (age range: 50–54)
					6.1 [5.8–6.5] (age range: 55–59)
60–69 years	8.1 ± 1.2	10.1 [6.4–18.8]	8.47 ± 1.09 [8.25–8.68]	6.8 ± 2.1	6.8 [6.5–7.0] (age range: 60–64)
≥70 years	9.5 ± 1.4 (age range: 70–79)	10.5 [6.3–18.1]	9.01 ± 2.00 [8.27–9.76]	7.9 ± 1.5 (age range: 70–79)	NA

NA—data not available, ^a^—95% confidence interval not available, ^b^—standard deviation not available.

**Table 2:** The main morphological characteristics of cardiovascular aging and their multimodality imaging features.

**Vascular aging**	
Arterial wall thickening	Increased carotid intima-media thickness
Arterial wall stiffening	Reduced aortic strain and distensibility, increased pulse–wave velocity
Atherosclerosis	Non-calcified and calcified atherosclerotic plaques
Arterial dilatation and elongation	Arterial tortuosity, increased diameter and length
**Cardiac aging**	
Myocardial hypertrophy	Increased left ventricular myocardial thickness and mass
Myocardial fibrosis	MRI tissue characterization required: prolonged myocardial T1 relaxation time, increased myocardial extracellular volume, late gadolinium enhancement
Valvular degeneration	Cusp thickening, stiffening, and calcification with reduced mobility
Diastolic dysfunction	Abnormal myocardial relaxation with impaired left ventricular filling
Coronary artery disease	CT: coronary artery calcification, coronary artery plaques with or without high-risk features, coronary artery stenosis
	MRI: myocardial ischemia (stress testing), regional wall motion abnormalities (cine imaging), ischemic pattern of late gadolinium enhancement

**Table 3:** Imaging features, indications, advantages, disadvantages, and restrictions for the use of different non-invasive imaging modalities in advanced CVD risk assessment.

	Imaging Features	Indications	Advantages	Disadvantages and Restrictions
Ultrasonography and echocardiography	Carotid imaging (carotid intima-media thickness, atherosclerotic plaques) Cardiac morphology and function Aortic stiffness (pulse wave velocity, aortic strain, and distensibility)	First-line imaging modality for assessment of carotid arteries First-line imaging modality for cardiac morphology and function assessment	Inexpensive Easily available Portable No ionizing radiation	Limited acoustic window Very limited possibilities for myocardial tissue characterization
Computed tomography	Coronary artery calcium (CAC) scoring Coronary CT angiography (CCTA): coronary stenosis and plaque assessment Aortic morphology and stiffness (aortic strain and distensibility)	Improvements in risk classification around treatment decision thresholds (CAC scoring) First-line imaging modality in patients with suspicious chronic coronary syndrome (CCTA) First-line imaging modality for acute aortic syndrome, commonly used for follow-up of aortic diseases (especially in older individuals)	Fast performance Detailed depiction of coronary anatomy (plaque morphology, luminal stenosis) and valvular morphology Method of choice for planning aortic interventional or surgical procedures	Ionizing radiation Use of iodine contrast agents High costs Imaging artifacts (motion artifacts, blooming artifacts) Limited functional information
Magnetic resonance imaging	Cardiac morphology and function Aortic morphology and stiffness (pulse wave velocity, aortic strain, and distensibility)	Imaging modality of choice for advanced assessment of cardiac morphology and function Non-invasive myocardial ischemia testing (stress test) Commonly used for follow-up of aortic diseases (especially in children and younger individuals)	Non-invasive myocardial tissue characterization Image acquisition in any desired plane The gold standard for assessment of myocardial mass, ventricular volumes, and ejection fraction	High costs Limited availability Long-lasting examinations Contraindications (ferromagnetic foreign bodies, non-conditional cardiac implantable electronic devices)

## Data Availability

No new data were created or analyzed in this study. Data sharing is not applicable to this article.

## References

[1] Cagigas M.L., Twigg S.M., Fontana L. (2024). Ten tips for promoting cardiometabolic health and slowing cardiovascular aging. Eur. Heart J..

[2] Tabaei B.P., Chamany S., Perlman S., Thorpe L., Bartley K., Wu W.Y. (2019). Heart Age, Cardiovascular Disease Risk, and Disparities by Sex and Race/Ethnicity Among New York City Adults. Public Health Rep..

[3] Visseren F.L.J., Mach F., Smulders Y.M., Carballo D., Koskinas K.C., Bäck M., Benetos A., Biffi A., Boavida J.M., Capodanno D. (2021). 2021 ESC Guidelines on cardiovascular disease prevention in clinical practice. Eur. Heart J..

[4] Terentes-Printzios D., Vlachopoulos C., Xaplanteris P., Ioakeimidis N., Aznaouridis K., Baou K., Kardara D., Georgiopoulos G., Georgakopoulos C., Tousoulis D. (2017). Cardiovascular Risk Factors Accelerate Progression of Vascular Aging in the General Population: Results From the CRAVE Study (Cardiovascular Risk Factors Affecting Vascular Age). Hypertension.

[5] World Health Organization Ageing and Health. https://www.who.int/news-room/fact-sheets/detail/ageing-and-health.

[6] Abdellatif M., Rainer P.P., Sedej S., Kroemer G. (2023). Hallmarks of cardiovascular ageing. Nat. Rev. Cardiol..

[7] Raisi-Estabragh Z., Szabo L., Schuermans A., Salih A.M., Chin C.W.L., Vágó H., Altmann A., Ng F.S., Garg P., Pavanello S. (2024). Noninvasive Techniques for Tracking Biological Aging of the Cardiovascular System: JACC Family Series. JACC Cardiovasc. Imaging.

[8] Knuuti J., Wijns W., Saraste A., Capodanno D., Barbato E., Funck-Brentano C., Prescott E., Storey R.F., Deaton C., Cuisset T. (2020). 2019 ESC Guidelines for the diagnosis and management of chronic coronary syndromes. Eur. Heart J..

[9] Gulati M., Levy P.D., Mukherjee D., Amsterdam E., Bhatt D.L., Birtcher K.K., Blankstein R., Boyd J., Bullock-Palmer R.P., Conejo T. (2021). 2021 AHA/ACC/ASE/CHEST/SAEM/SCCT/SCMR Guideline for the Evaluation and Diagnosis of Chest Pain: A Report of the American College of Cardiology/American Heart Association Joint Committee on Clinical Practice Guidelines. Circulation.

[10] Peralta C.A., Adeney K.L., Shlipak M.G., Jacobs D., Duprez D., Bluemke D., Polak J., Psaty B., Kestenbaum B.R. (2010). Structural and functional vascular alterations and incident hypertension in normotensive adults: The Multi-Ethnic Study of Atherosclerosis. Am. J. Epidemiol..

[11] Bruno R.M., Bianchini E., Faita F., Taddei S., Ghiadoni L. (2014). Intima media thickness, pulse wave velocity, and flow mediated dilation. Cardiovasc. Ultrasound.

[12] Lakatta E.G. (2015). So! What’s aging? Is cardiovascular aging a disease?. J. Mol. Cell. Cardiol..

[13] Lakatta E.G., Levy D. (2003). Arterial and cardiac aging: Major shareholders in cardiovascular disease enterprises: Part I: Aging arteries: A “set up” for vascular disease. Circulation.

[14] Kozakova M., Palombo C. (2021). Vascular Ageing and Aerobic Exercise. Int. J. Environ. Res. Public Health.

[15] Lan Y.S., Khong T.K., Yusof A. (2023). Effect of Exercise on Arterial Stiffness in Healthy Young, Middle-Aged and Older Women: A Systematic Review. Nutrients.

[16] Kobayashi R., Asaki K., Hashiguchi T., Negoro H. (2022). Effect of aerobic exercise training frequency on arterial stiffness in middle-aged and elderly females. J. Phys. Ther. Sci..

[17] O’Rourke M., Farnsworth A., O’Rourke J. (2008). Aortic dimensions and stiffness in normal adults. JACC Cardiovasc. Imaging.

[18] O’Rourke M.F., Nichols W.W. (2005). Aortic diameter, aortic stiffness, and wave reflection increase with age and isolated systolic hypertension. Hypertension.

[19] Sugawara J., Hayashi K., Yokoi T., Tanaka H. (2008). Age-associated elongation of the ascending aorta in adults. JACC Cardiovasc. Imaging.

[20] Adriaans B.P., Heuts S., Gerretsen S., Cheriex E.C., Vos R., Natour E., Maessen J.G., Sardari Nia P., Crijns H.J.G.M., Wildberger J.E. (2018). Aortic elongation part I: The normal aortic ageing process. Heart.

[21] O’Rourke M.F., Hashimoto J. (2007). Mechanical factors in arterial aging: A clinical perspective. J. Am. Coll. Cardiol..

[22] Lanzer P., Boehm M., Sorribas V., Thiriet M., Janzen J., Zeller T., St Hilaire C., Shanahan C. (2014). Medial vascular calcification revisited: Review and perspectives. Eur. Heart J..

[23] Oberoi S., Schoepf U.J., Meyer M., Henzler T., Rowe G.W., Costello P., Nance J.W. (2013). Progression of arterial stiffness and coronary atherosclerosis: Longitudinal evaluation by cardiac CT. AJR Am. J. Roentgenol..

[24] Cheng D.C.Y., Climie R.E., Shu M., Grieve S.M., Kozor R., Figtree G.A. (2023). Vascular aging and cardiovascular disease: Pathophysiology and measurement in the coronary arteries. Front. Cardiovasc. Med..

[25] Laurent S., Boutouyrie P., Cunha P.G., Lacolley P., Nilsson P.M. (2019). Concept of Extremes in Vascular Aging. Hypertension.

[26] Wilkinson I.B., Mäki-Petäjä K.M., Mitchell G.F. (2020). Uses of Arterial Stiffness in Clinical Practice. Arter. Thromb. Vasc. Biol..

[27] Mancia G., Kreutz R., Brunström M., Burnier M., Grassi G., Januszewicz A., Muiesan M.L., Tsioufis K., Agabiti-Rosei E., Algharably E.A.E. (2023). 2023 ESH Guidelines for the management of arterial hypertension the Task Force for the management of arterial hypertension of the European Society of Hypertension: Endorsed by the International Society of Hypertension (ISH) and the European Renal Association (ERA). J. Hypertens..

[28] Arnett D.K., Blumenthal R.S., Albert M.A., Buroker A.B., Goldberger Z.D., Hahn E.J., Himmelfarb C.D., Khera A., Lloyd-Jones D., McEvoy J.W. (2019). 2019 ACC/AHA Guideline on the Primary Prevention of Cardiovascular Disease: A Report of the American College of Cardiology/American Heart Association Task Force on Clinical Practice Guidelines. Circulation.

[29] Parikh J.D., Hollingsworth K.G., Kunadian V., Blamire A., MacGowan G.A. (2016). Measurement of pulse wave velocity in normal ageing: Comparison of Vicorder and magnetic resonance phase contrast imaging. BMC Cardiovasc. Disord..

[30] Baier D., Teren A., Wirkner K., Loeffler M., Scholz M. (2018). Parameters of pulse wave velocity: Determinants and reference values assessed in the population-based study LIFE-Adult. Clin. Res. Cardiol..

[31] Díaz A., Galli C., Tringler M., Ramírez A., Cabrera Fischer E.I. (2014). Reference values of pulse wave velocity in healthy people from an urban and rural argentinean population. Int. J. Hypertens..

[32] van Hout M.J., Dekkers I.A., Westenberg J.J., Schalij M.J., Widya R.L., de Mutsert R., Rosendaal F.R., de Roos A., Jukema J.W., Scholte A.J. (2021). Normal and reference values for cardiovascular magnetic resonance-based pulse wave velocity in the middle-aged general population. J. Cardiovasc. Magn. Reason..

[33] Kwak H.B. (2013). Effects of aging and exercise training on apoptosis in the heart. J. Exerc. Rehabil..

[34] Pokharel P., Bella J.N. (2013). Regression of left ventricular hypertrophy: Lessons from clinical trials. OA Evid.-Based Med..

[35] Lakatta E.G., Levy D. (2003). Arterial and cardiac aging: Major shareholders in cardiovascular disease enterprises: Part II: The aging heart in health: Links to heart disease. Circulation.

[36] Shioi T., Inuzuka Y. (2012). Aging as a substrate of heart failure. J. Cardiol..

[37] Forman D.E., de Lemos J.A., Shaw L.J., Reuben D.B., Lyubarova R., Peterson E.D., Spertus J.A., Zieman S., Salive M.E., Rich M.W. (2020). Cardiovascular Biomarkers and Imaging in Older Adults: JACC Council Perspectives. J. Am. Coll. Cardiol..

[38] Lloyd-Jones D.M., Wang T.J., Leip E.P., Larson M.G., Levy D., Vasan R.S., D’Agostino R.B., Massaro J.M., Beiser A., Wolf P.A. (2004). Lifetime risk for development of atrial fibrillation: The Framingham Heart Study. Circulation.

[39] Ji H., Kwan A.C., Chen M.T., Ouyang D., Ebinger J.E., Bell S.P., Niiranen T.J., Bello N.A., Cheng S. (2022). Sex Differences in Myocardial and Vascular Aging. Circ. Res..

[40] Kuklina E.V. (2010). Assessing and managing risk for cardiovascular disease: A worldwide perspective. N. Am. J. Med. Sci..

[41] Rajendran A., Minhas A.S., Kazzi B., Varma B., Choi E., Thakkar A., Michos E.D. (2023). Sex-specific differences in cardiovascular risk factors and implications for cardiovascular disease prevention in women. Atherosclerosis.

[42] Jeffers A.M., Glantz S., Byers A.L., Keyhani S. (2024). Association of Cannabis Use With Cardiovascular Outcomes Among US Adults. J. Am. Heart Assoc..

[43] Hageman S.H.J., Petitjaen C., Pennells L., Kaptoge S., Pajouheshnia R., Tillmann T., Blaha M.J., McClelland R.L., Matsushita K., Nambi V. (2023). Improving 10-year cardiovascular risk prediction in apparently healthy people: Flexible addition of risk modifiers on top of SCORE2. Eur. J. Prev. Cardiol..

[44] Vecsey-Nagy M., Szilveszter B., Kolossváry M., Boussoussou M., Vattay B., Merkely B., Maurovich-Horvat P., Radovits T., Nemcsik J. (2022). Correlation between Coronary Artery Calcium- and Different Cardiovascular Risk Score-Based Methods for the Estimation of Vascular Age in Caucasian Patients. J. Clin. Med..

[45] Li C., Liu X., Shen P., Sun Y., Zhou T., Chen W., Chen Q., Lin H., Tang X., Gao P. (2024). Improving cardiovascular risk prediction through machine learning modelling of irregularly repeated electronic health records. Eur. Heart J. Digit. Health.

[46] Stein J.H., Korcarz C.E., Hurst R.T., Lonn E., Kendall C.B., Mohler E.R., Najjar S.S., Rembold C.M., Post W.S., American Society of Echocardiography Carotid Intima-Media Thickness Task Force (2008). Use of carotid ultrasound to identify subclinical vascular disease and evaluate cardiovascular disease risk: A consensus statement from the American Society of Echocardiography Carotid Intima-Media Thickness Task Force. Endorsed by the Society for Vascular Medicine. J. Am. Soc. Echocardiogr..

[47] Folsom A.R., Kronmal R.A., Detrano R.C., O’Leary D.H., Bild D.E., Bluemke D.A., Budoff M.J., Liu K., Shea S., Szklo M. (2008). Coronary artery calcification compared with carotid intima-media thickness in the prediction of cardiovascular disease incidence: The Multi-Ethnic Study of Atherosclerosis (MESA). Arch. Intern. Med..

[48] Den Ruijter H.M., Peters S.A., Anderson T.J., Britton A.R., Dekker J.M., Eijkemans M.J., Engstrom G., Evans G.W., de Graaf J., Grobbee D.E. (2012). Common carotid intima-media thickness measurements in cardiovascular risk prediction: A meta-analysis. JAMA.

[49] Raitakari O.T., Magnussen C.G., Juonala M., Kartiosuo N., Pahkala K., Rovio S., Koskinen J.S., Mykkänen J., Laitinen T.P., Kähönen M. (2024). Subclinical atherosclerosis in young adults predicting cardiovascular disease: The Cardiovascular Risk in Young Finns Study. Atherosclerosis.

[50] Ganau A., Orrù M., Floris M., Saba P.S., Loi F., Sanna G.D., Marongiu M., Balaci L., Curreli N., Ferreli L.A.P. (2024). Echocardiographic heart ageing patterns predict cardiovascular and non-cardiovascular events and reflect biological age: The SardiNIA study. Eur. J. Prev. Cardiol..

[51] Leong D.P., Delgado V., Bax J.J. (2012). Imaging for atrial fibrillation. Curr. Probl. Cardiol..

[52] Cho J.Y., Kim K.H. (2016). Evaluation of Arterial Stiffness by Echocardiography: Methodological Aspects. Chonnam Med. J..

[53] Agatston A.S., Janowitz W.R., Hildner F.J., Zusmer N.R., Viamonte M., Detrano R. (1990). Quantification of coronary artery calcium using ultrafast computed tomography. J. Am. Coll. Cardiol..

[54] Budoff M.J., Achenbach S., Blumenthal R.S., Carr J.J., Goldin J.G., Greenland P., Guerci A.D., Lima J.A., Rader D.J., Rubin G.D. (2006). Assessment of coronary artery disease by cardiac computed tomography: A scientific statement from the American Heart Association Committee on Cardiovascular Imaging and Intervention, Council on Cardiovascular Radiology and Intervention, and Committee on Cardiac Imaging, Council on Clinical Cardiology. Circulation.

[55] Alluri K., Joshi P.H., Henry T.S., Blumenthal R.S., Nasir K., Blaha M.J. (2015). Scoring of coronary artery calcium scans: History, assumptions, current limitations, and future directions. Atherosclerosis.

[56] Hecht H.S., Blaha M.J., Kazerooni E.A., Cury R.C., Budoff M., Leipsic J., Shaw L. (2018). CAC-DRS: Coronary Artery Calcium Data and Reporting System. An expert consensus document of the Society of Cardiovascular Computed Tomography (SCCT). J. Cardiovasc. Comput. Tomogr..

[57] Greenland P., Alpert J.S., Beller G.A., Benjamin E.J., Budoff M.J., Fayad Z.A., Foster E., Hlatky M.A., Hodgson J.M., Kushner F.G. (2010). 2010 ACCF/AHA guideline for assessment of cardiovascular risk in asymptomatic adults: A report of the American College of Cardiology Foundation/American Heart Association Task Force on Practice Guidelines. Circulation.

[58] Alani A., Budoff M.J. (2014). Coronary calcium scoring and computed tomography angiography: Current indications, future applications. Coron. Artery Dis..

[59] van Praagh G.D., Wang J., van der Werf N.R., Greuter M.J.W., Mastrodicasa D., Nieman K., van Hamersvelt R.W., Oostveen L.J., de Lange F., Slart R.H.J.A. (2022). Coronary Artery Calcium Scoring: Toward a New Standard. Investig. Radiol..

[60] Shah N.R., Coulter S.A. (2012). An evidence-based guide for coronary calcium scoring in asymptomatic patients without coronary heart disease. Tex. Heart Inst. J..

[61] Greenland P., Bonow R.O., Brundage B.H., Budoff M.J., Eisenberg M.J., Grundy S.M., Lauer M.S., Post W.S., Raggi P., Redberg R.F. (2007). ACCF/AHA 2007 clinical expert consensus document on coronary artery calcium scoring by computed tomography in global cardiovascular risk assessment and in evaluation of patients with chest pain: A report of the American College of Cardiology Foundation Clinical Expert Consensus Task Force (ACCF/AHA Writing Committee to Update the 2000 Expert Consensus Document on Electron Beam Computed Tomography). Circulation.

[62] Sheppard J.P., Lakshmanan S., Lichtenstein S.J., Budoff M.J., Roy S.K. (2022). Age and the power of zero CAC in cardiac risk assessment: Overview of the literature and a cautionary case. Br. J. Cardiol..

[63] Grandhi G.R., Mirbolouk M., Dardari Z.A., Al-Mallah M.H., Rumberger J.A., Shaw L.J., Blankstein R., Miedema M.D., Berman D.S., Budoff M.J. (2020). Interplay of Coronary Artery Calcium and Risk Factors for Predicting CVD/CHD Mortality: The CAC Consortium. JACC Cardiovasc. Imaging.

[64] Whelton S.P., Blaha M.J. (2023). Coronary artery calcium: From risk prediction to treatment allocation and clinical trials. Heart.

[65] Polonsky T.S., McClelland R.L., Jorgensen N.W., Bild D.E., Burke G.L., Guerci A.D., Greenland P. (2010). Coronary artery calcium score and risk classification for coronary heart disease prediction. JAMA.

[66] Sandoval Y., Bielinski S.J., Daniels L.B., Blaha M.J., Michos E.D., DeFilippis A.P., Szklo M., deFilippi C., Larson N.B., Decker P.A. (2020). Atherosclerotic Cardiovascular Disease Risk Stratification Based on Measurements of Troponin and Coronary Artery Calcium. J. Am. Coll. Cardiol..

[67] Miname M.H., Bittencourt M.S., Pereira A.C., Jannes C.E., Krieger J.E., Nasir K., Santos R.D. (2020). Vascular age derived from coronary artery calcium score on the risk stratification of individuals with heterozygous familial hypercholesterolaemia. Eur. Heart J. Cardiovasc. Imaging.

[68] Mamudu H.M., Paul T.K., Veeranki S.P., Budoff M. (2014). The effects of coronary artery calcium screening on behavioral modification, risk perception, and medication adherence among asymptomatic adults: A systematic review. Atherosclerosis.

[69] Peters S.A., den Ruijter H.M., Bots M.L., Moons K.G. (2012). Improvements in risk stratification for the occurrence of cardiovascular disease by imaging subclinical atherosclerosis: A systematic review. Heart.

[70] Golub I.S., Termeie O.G., Kristo S., Schroeder L.P., Lakshmanan S., Shafter A.M., Hussein L., Verghese D., Aldana-Bitar J., Manubolu V.S. (2023). Major Global Coronary Artery Calcium Guidelines. JACC Cardiovasc. Imaging.

[71] MESA Risk Score and Coronary Age Calculator. http://www.mesa-nhlbi.org/Calcium/ArterialAge.aspx.

[72] Onnis C., Muscogiuri G., Cademartiri F., Fanni D., Faa G., Gerosa C., Mannelli L., Suri J.S., Sironi S., Montisci R. (2023). Non-invasive coronary imaging in elderly population. Eur. J. Radiol..

[73] Cho I., Al’Aref S.J., Berger A., Hartaigh B.Ó., Gransar H., Valenti V., Lin F.Y., Achenbach S., Berman D.S., Budoff M.J. (2018). Prognostic value of coronary computed tomographic angiography findings in asymptomatic individuals: A 6-year follow-up from the prospective multicentre international CONFIRM study. Eur. Heart J..

[74] Meah M.N., Maurovich-Horvat P., Williams M.C., Newby D.E. (2022). Debates in cardiac CT: Coronary CT angiography is the best test in asymptomatic patients. J. Cardiovasc. Comput. Tomogr..

[75] Dell’Aversana S., Ascione R., De Giorgi M., De Lucia D.R., Cuocolo R., Boccalatte M., Sibilio G., Napolitano G., Muscogiuri G., Sironi S. (2022). Dual-Energy CT of the Heart: A Review. J. Imaging.

[76] Cetin T., Kantarci M., Irgul B., Aydin S., Aydin F., Koseturk T., Levent A. (2023). Quadruple-Rule-Out Computed Tomography Angiography (QRO-CT): A Novel Dual-Energy Computed Tomography Technique for the Diagnostic Work-Up of Acute Chest Pain. Diagnostics.

[77] Pack J.D., Xu M., Wang G., Baskaran L., Min J., De Man B. (2022). Cardiac CT blooming artifacts: Clinical significance, root causes and potential solutions. Vis. Comput. Ind. Biomed. Art..

[78] Tonet E., Amantea V., Lapolla D., Assabbi P., Boccadoro A., Berloni M.L., Micillo M., Marchini F., Chiarello S., Cossu A. (2023). Cardiac Computed Tomography in Monitoring Revascularization. J. Clin. Med..

[79] Bianchini E., Lønnebakken M.T., Wohlfahrt P., Piskin S., Terentes-Printzios D., Alastruey J., Guala A. (2023). Magnetic Resonance Imaging and Computed Tomography for the Noninvasive Assessment of Arterial Aging: A Review by the VascAgeNet COST Action. J. Am. Heart Assoc..

[80] Mileto A., Heye T.J., Makar R.A., Hurwitz L.M., Marin D., Boll D.T. (2016). Regional Mapping of Aortic Wall Stress by Using Deformable, Motion-coherent Modeling based on Electrocardiography-gated Multidetector CT Angiography: Feasibility Study. Radiology.

[81] Kersten J., Hackenbroch C., Bouly M., Tyl B., Bernhardt P. (2022). What Is Normal for an Aging Heart?: A Prospective CMR Cohort Study. J. Cardiovasc. Imaging.

[82] Paterson I., Mielniczuk L.M., O’Meara E., So A., White J.A. (2013). Imaging heart failure: Current and future applications. Can. J. Cardiol..

[83] Armstrong A.C., Gidding S., Gjesdal O., Wu C., Bluemke D.A., Lima J.A. (2012). LV mass assessed by echocardiography and CMR, cardiovascular outcomes, and medical practice. JACC Cardiovasc. Imaging.

[84] Raisi-Estabragh Z., Salih A., Gkontra P., Atehortúa A., Radeva P., Boscolo Galazzo I., Menegaz G., Harvey N.C., Lekadir K., Petersen S.E. (2022). Estimation of biological heart age using cardiovascular magnetic resonance radiomics. Sci. Rep..

[85] Shah M., de A Inácio M.H., Lu C., Schiratti P.R., Zheng S.L., Clement A., de Marvao A., Bai W., King A.P., Ware J.S. (2023). Environmental and genetic predictors of human cardiovascular ageing. Nat. Commun..

[86] Koh A.S., Gao F., Leng S., Kovalik J.P., Zhao X., Tan R.S., Fridianto K.T., Ching J., Chua S.J., Yuan J.M. (2018). Dissecting Clinical and Metabolomics Associations of Left Atrial Phasic Function by Cardiac Magnetic Resonance Feature Tracking. Sci. Rep..

[87] Salih A.M., Pujadas E.R., Campello V.M., McCracken C., Harvey N.C., Neubauer S., Lekadir K., Nichols T.E., Petersen S.E., Raisi-Estabragh Z. (2023). Image-Based Biological Heart Age Estimation Reveals Differential Aging Patterns Across Cardiac Chambers. J. Magn. Reson. Imaging.

[88] Rosmini S., Bulluck H., Captur G., Treibel T.A., Abdel-Gadir A., Bhuva A.N., Culotta V., Merghani A., Fontana M., Maestrini V. (2018). Myocardial native T1 and extracellular volume with healthy ageing and gender. Eur. Heart J. Cardiovasc. Imaging.

[89] Neilan T.G., Coelho-Filho O.R., Shah R.V., Abbasi S.A., Heydari B., Watanabe E., Chen Y., Mandry D., Pierre-Mongeon F., Blankstein R. (2013). Myocardial extracellular volume fraction from T1 measurements in healthy volunteers and mice: Relationship to aging and cardiac dimensions. JACC Cardiovasc. Imaging.

[90] Liu C.Y., Liu Y.C., Wu C., Armstrong A., Volpe G.J., van der Geest R.J., Liu Y., Hundley W.G., Gomes A.S., Liu S. (2013). Evaluation of age-related interstitial myocardial fibrosis with cardiac magnetic resonance contrast-enhanced T1 mapping: MESA (Multi-Ethnic Study of Atherosclerosis). J. Am. Coll. Cardiol..

[91] Gilstrap L.G., Dominici F., Wang Y., El-Sady M.S., Singh A., Di Carli M.F., Falk R.H., Dorbala S. (2019). Epidemiology of Cardiac Amyloidosis-Associated Heart Failure Hospitalizations Among Fee-for-Service Medicare Beneficiaries in the United States. Circ. Heart Fail..

[92] Li W., Uppal D., Wang Y.C., Xu X., Kokkinidis D.G., Travin M.I., Tauras J.M. (2021). Nuclear Imaging for the Diagnosis of Cardiac Amyloidosis in 2021. Diagnostics.

[93] Marwick T.H., Raman S.V., Carrio I., Bax J.J. (2010). Recent developments in heart failure imaging. JACC Cardiovasc. Imaging.

[94] Dogui A., Redheuil A., Lefort M., DeCesare A., Kachenoura N., Herment A., Mousseaux E. (2011). Measurement of aortic arch pulse wave velocity in cardiovascular MR: Comparison of transit time estimators and description of a new approach. J. Magn. Reson. Imaging.

[95] Soulat G., Gencer U., Kachenoura N., Villemain O., Messas E., Boutouyrie P., Laurent S., Mousseaux E. (2020). Changes in segmental pulse wave velocity of the thoracic aorta with age and left ventricular remodelling. An MRI 4D flow study. J. Hypertens..

[96] Loose S., Solou D., Strecker C., Hennemuth A., Hüllebrand M., Grundmann S., Asmussen A., Treppner M., Urbach H., Harloff A. (2023). Characterization of aortic aging using 3D multi-parametric MRI-long-term follow-up in a population study. Sci. Rep..

[97] Redheuil A., Kachenoura N., Bollache E., Yu W.C., Opdahl A., Decesare A., Mousseaux E., Bluemke D., Lima J.A.C. (2019). Left ventricular and proximal aorta coupling in magnetic resonance imaging: Aging together?. Am. J. Physiol. Heart Circ. Physiol..

[98] Garrido-Oliver J., Aviles J., Córdova M.M., Dux-Santoy L., Ruiz-Muñoz A., Teixido-Tura G., Maso Talou G.D., Morales Ferez X., Jiménez G., Evangelista A. (2022). Machine learning for the automatic assessment of aortic rotational flow and wall shear stress from 4D flow cardiac magnetic resonance imaging. Eur. Radiol..

[99] Cecelja M., Ruijsink B., Puyol-Antón E., Li Y., Godwin H., King A.P., Razavi R., Chowienczyk P. (2022). Aortic Distensibility Measured by Automated Analysis of Magnetic Resonance Imaging Predicts Adverse Cardiovascular Events in UK Biobank. J. Am. Heart Assoc..

[100] Hrabak-Paar M., Kircher A., Al Sayari S., Kopp S., Santini F., Schmieder R.E., Kachenoura N., Yates D., Langenickel T., Bremerich J. (2020). Variability of MRI Aortic Stiffness Measurements in a Multicenter Clinical Trial Setting: Intraobserver, Interobserver, and Intracenter Variability of Pulse Wave Velocity and Aortic Strain Measurement. Radiol. Cardiothorac. Imaging.

[101] Spronck B., Terentes-Printzios D., Avolio A.P., Boutouyrie P., Guala A., Jerončić A., Laurent S., Barbosa E.C.D., Baulmann J., Chen C.H. (2024). 2024 Recommendations for Validation of Noninvasive Arterial Pulse Wave Velocity Measurement Devices. Hypertension.

[102] Herment A., Kachenoura N., Lefort M., Bensalah M., Dogui A., Frouin F., Mousseaux E., De Cesare A. (2010). Automated segmentation of the aorta from phase contrast MR images: Validation against expert tracing in healthy volunteers and in patients with a dilated aorta. J. Magn. Reson. Imaging.

[103] Xiang S., Li N., Wang Y., Zhou S., Wei J., Li S. (2024). Automatic Delineation of the 3D Left Atrium From LGE-MRI: Actor-Critic Based Detection and Semi-Supervised Segmentation. IEEE J. Biomed. Health Inform..

[104] Li J., Wu Q., Wang Y., Zhou S., Zhang L., Wei J., Zhao D. (2024). DiffCAS: Diffusion based multi-attention network for segmentation of 3D coronary artery from CT angiography. Signal Image Video Process..

[105] Zhao C., Xiang S., Wang Y., Cai Z., Shen J., Zhou S., Zhao D., Su W., Guo S., Li S. (2023). Context-aware network fusing transformer and V-Net for semi-supervised segmentation of 3D left atrium. Expert Syst. Appl..

